# Spatial Modeling of Equine Herpesviruses 1 (EHVs-1) Risks in Kazakhstan Using 2017–2024 Surveillance Data

**DOI:** 10.1155/tbed/5536099

**Published:** 2025-08-21

**Authors:** Yersyn Mukhanbetkaliyev, Gulzhan Yessembekova, Aizada Mukhanbetkaliyeva, Botakoz Akmambayeva, Ablaikhan Kadyrov, Rashit Uskenov, Saule Bostanova, Alibek Ashirbek, Fedor Korennoy, Sarsenbay Abdrakhmanov

**Affiliations:** ^1^S. Seifullin Kazakh Agro Technical Research University, Astana, Kazakhstan; ^2^Federal Center for Animal Health (FGBI ARRIAH), Vladimir, Russia; ^3^Federal Research Center for Virology and Microbiology–Branch in Nizhny Novgorod, Nizhny Novgorod, Russia

**Keywords:** EHV-1, Equine herpesviruses, forest-based regression, horse breeding, Kazakhstan, risk mapping, suitability

## Abstract

Equine herpesvirus 1 (EHV-1) is one of the most dangerous viral diseases affecting ungulates, and is characterized by a wide range of clinical manifestations in horses, including rhinopneumonia, abortion, neonatal death, and myeloencephalopathy. It is well known for causing mass abortions in mares and respiratory diseases in young animals. Once introduced into a horse breeding farm of any type, EHV-1 tends to establish as a persistent infection. The disease is reported on nearly all continents and causes substantial annual economic losses to horse breeding operations. In Kazakhstan, 34 EHV-1 outbreaks were recorded between 2017 and 2024. The objective of our study was to identify potential risk factors associated with the presence of EHV-1 within the study area. We employed a forest-based classification and regression approach to explore a set of sociodemographic, environmental, and transportation-related factors associated with the presence or absence of EHV-1 at the level of administrative regions. A standard set of explanatory variables was supplemented with horse population density, derived from demographic data of horse-breeding farms obtained through a nationwide survey. Modeling results indicated that the most significant factor influencing EHV-1 presence was the average wind speed in January, followed by road density, the number of horse farms, and the number of livestock-related facilities targeted for surveillance. Horse population density was found to be among the least significant variable in the model. The resulting risk map highlights areas with a higher suitability for EHV-1 emergence, primarily located in regions with moderate-to-high horse population densities and characterized by steppe- and grassland-type landscapes, which are predominantly found in the northern, central, and south-western parts of Kazakhstan. These findings can serve as a foundation for further investigation into the spatial patterns of EHV-1 in the country and for enhancing veterinary surveillance and control measures.

## 1. Introduction

Horse breeding in Kazakhstan is one of the fastest-growing sectors of animal husbandry. This is influenced by important factors for the growth of livestock, such as extensive pastures, centuries-old experience of horse breeding in the country, the reverent attitude of the population of the Republic toward these animals, the presence of breeds adapted to the difficult climatic conditions of Kazakhstan, and the nationwide passion for national equestrian sports [1–3]. Given the national peculiarity of the prerogative for horse meat, there will always be a demand for equine products in Kazakhstan. Also, horse breeding is important for Kazakhstan not only to meet the needs of the meat and dairy sectors, namely the production of kumys and saumal. Over the past 15–20 years, the number of horses in the republic has increased by more than 1.7 million heads. In 2003, the number of horses slightly exceeded 1 million. Then their number steadily increased by about 100,000 annually, and by the end of 2019, the horse breeders of Kazakhstan totaled the number of animals to 2.9 million heads. According to data from the Food and Agriculture Organization of the UN (FAO), Kazakhstan occupies the 9th place in the world in terms of horse numbers, with a population size exceeding 2.9 million heads. Today, according to data from the authorized body of statistics in the Republic of Kazakhstan (RK), 3.4 million horses were recorded in the republic https://stat.gov.kz/official/industry/14/statistic/7 (as of April 1, 2023). At the same time, infectious diseases present one of the constraining factors for developing horse breeding in the country, as in other livestock industries. In this regard, herpesvirus infections, primarily Equine herpesvirus-1 ([EHV-1], infectious rhinopneumonia—viral abortion of horses), pose a constant threat to horse breeding in many countries of the world.

EHV-1 is a cause of dangerous viral diseases of ungulates. It is characterized by a significant variety of clinical signs in horses, such as rhinopneumonia, abortion, neonatal death, and myeloencephalopathy [4–6]. The severity of EHV-1 infection depends on several factors, including gender, age, physiological status (pregnancy), host immunity, and the properties of viral strains. Some EHV-1 strains are highly virulent and cause disease with more severe symptoms [7].

EHV-1 causes significant economic damage to horse breeding, which consists of the loss of the reproductive ability of young horses, the failure to give brith to offspring, the culling of horses valuable in breeding, and the implementation of veterinary and sanitary measures. After emerging in a horse farm of any type (stud farm, breeding farm, racetrack, equestrian complex, etc.), EHV-1 takes on the character of a stationary infection [8, 9]. At the same time, the disease that emerges in previously prosperous farms often takes the form of an acute mass outbreak, during which all livestock are infected. From 40% to 60% of foaled mares (sometimes the ratio reaches 90%) undergo abortions. Such explosive abortions are known as “storm abortions” [10].

The acute course of infection alternates with periods of atypical manifestations of the disease, making diagnosis extremely difficult [11]. The respiratory strains of rhinopneumonia mainly affect the respiratory organs, whereas the fetal strains cause abortion in foal. However, in a given population of horses (a separate horse farm, herd, and horse breeding farm), the existence and independent reproduction of both types of EHV-1 (respiratory and fetal) are possible. Each cause outbreaks of the disease that can overlap with one another [10].

The epizootic situation of EHV-1 in many countries throughout the world remains tense and ambiguous. The disease is registered on almost all continents, and it annually results in significant economic losses for horse breeding farms [12]. In recent years, a significant increase in the incidence of EHV infection has been observed in countries with developed horse breeding (the USA, Canada, and the EU). In addition to direct economic losses for horse farms, outbreaks of EHV can disrupt important economic sectors, such as horse racing. The manifestations of EHV-1 infection in different countries vary from isolated sporadic cases to infection in 90% of the herd. Mortality also varies during outbreaks and can reach 40%–50% [13, 14].

So, in 2017–2018, local outbreaks of EHV-1 infection in horses with signs of encephalomyelitis and atypical abortions were registered on the territory of the Russian Federation. In particular, annual outbreaks were observed in the Republic of Sakha (Yakutia), where the circulation of the EHV was recorded in 99 settlements of 16 uluses [15].

In China, there was an outbreak of EHV in or horses on the Chinese state horse farm Zhaosu, located on the border with Kazakhstan (Northern Xinjiang, China), when 43 out of 800 pregnant mares were aborted in January, 2021 [16].

In the USA, the cases of infected horses with the EHV-1 are a big problem, expressed by myeloencephalopathy with high mortality. The presence of horse rhinopneumonia was confirmed on horse breeding farms by the detection of EHV-1 latent DNA in the tissues of 71 (54%) of 132 mares. The presence of a relatively large biological reservoir of latent neuropathogenic EHV-1 potentially poses new threats to the emergence of infection [17, 18].

The study of EHV-1 in recent years in Kazakhstan has shown that the herpesvirus infections still exist in the republic. Thus, according to the official veterinary reports for 2017–2024, 34 foci of EHV-1 were registered across the territory of the republic. The circulation of the EHV-1 serotype virus was established in 52.5% of horses in Kazakhstan. Economic damage to horse breeding consists of the loss of reproductive capacity of young horses, the loss of offspring, and the culling of animals. In addition to direct damage, this infection creates obstacles to international trade and transportation of both horses and a large arsenal of goods controlled by the veterinary service. So, in 2010, during the epizootic in Kazakhstan, all the infected animals sharply reduced their marketable value. However, the epizootology of herpesvirus infection, taking into account regional peculiarities, has not been studied [19].

In this regard, regional studies of the epizootic process are of great importance in the system of measures aimed at the prevention and control of EHV-1. It will allow to study the peculiarities of the disease manifestation in a specific territory, in specific natural-geographical and socioeconomic conditions, with subsequent forecasting as a reliable foundation for managing the epizootic process.

## 2. Materials and Methods

### 2.1. Study Area

The research was conducted across the entire territory of the RK. The RK is a country in Central Asia, the ninth biggest country in the world with a population of 19,644,000 people. Administratively, the RK is divided into 17 first-level units (regions), three cities of republican significance, and 176 second-level units (municipalities or districts). The latter were used as modeling units in our study; their area varies between 282 and 128,663 km^2^.

### 2.2. Horse Population Data

**Figure 1:**
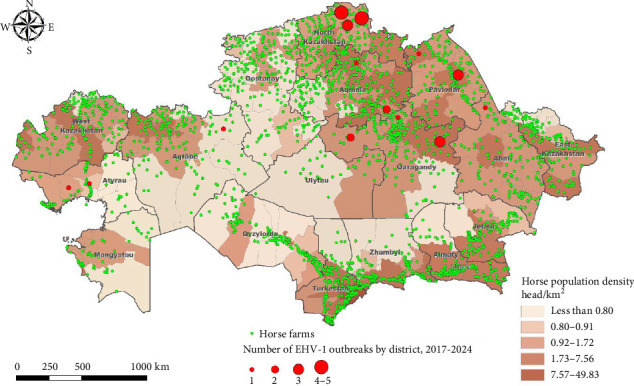
The 1st- and 2nd-level administrative divisions of Kazakhstan, the horse population density, the location of horse breeding farms, and EHV-1 affected districts during 2017–2024.

The database on the location of horse breeding farms in the RK was collected between 2019 and 2022 during the implementation of the scientific and technical program aimed at the development and creation of scientifically grounded “smart farms” in Kazakhstan. In total, the location of 2411 farms was recorded (Figure 1). The number of horses in farms varies between 1 and 30,783 head (the median value is 961 head). Based on this information, the number of horses was linked to the districts of the RK, and the average density of livestock was calculated, which ranged from 0 to 49.8 head/km^2^ (median value 1.37 head/km^2^). The district-level equine densities were further used for visualization and modeling (Figure 1).

### 2.3. Data on EHV-1 Cases in the RK

During the period between 2017 and 2024, 34 outbreaks of equine infectious rhinopneumonia virus type EHV-1 were recorded in the RK. Due to confidentiality of data, we did not have access to the exact location and attribution of infected farms and used only their yearly number (Table 1) and affected district instead (Figure 1). There is no information on the size of infected herds or the number of infected animals. The diagnosis was established by the Republican State Enterprise with the Right of Economic Use, the “National Reference Center for Veterinary Medicine,” under the Ministry of Agriculture of the RK. The state conducts an epizootological monitoring for EHV-1 country wise. Annually, 1500 susceptible animals are tested regardless of age, breed, and farm ownership type. The testing locations and sizes are defined by taking into account the current epidemic situation in the country and using a randomized approach in all settlements where horse farms are present. An enzyme-linked immunosorbent assay (ELISA) testing kit by Ingenaza (Spain) is used for serological monitoring (https://www.goldstandarddiagnostics.es/home-es/productos/veterinaria/elisa-y-tests-r%c3%a1pidos-veterinaria/equino/hve-rinoneumonitis-equina/ingezim-rinoneumonitis/). In the case of a positive sample, additional testing by PCR is applied to detect and confirm EHV-1.

### 2.4. Modeling Method

To reveal factors contributing to the RK districts' vulnerability to the EHV-1 virus and to obtain a risk map, a modeling method based on the forest-based classification and regression was employed. This approach classifies the RK districts as per infection status in regard to a set of risk factors, which potentially contribute to the probability of the infection emergence and transmission. A choice of this method is justified by the limited information on the attribution and location of infected farms that only leaves one reliable binary indicator of the epidemic status: infected or not infected [20, 21]. Other advantages of the applied method include the interpretability of its results, which facilitates the identification of variables that have the greatest influence on the distribution of the studied phenomenon. The method is also insensitive to the scaling of feature values, making it suitable for datasets with heterogeneous and multiscale variables. Additionally, it demonstrates robustness to noisy data, enhancing its reliability in real-world applications. In our case, the use of traditional regression models would be less appropriate due to the relatively small number of infected districts (17 out of 174) and the presence of a set of heterogeneous and multiscale factors [22, 23]. Briefly, this approach creates many decision trees, called an ensemble or a forest, that are used for prediction. Each tree generates its own prediction and is used as part of a voting scheme to make final predictions. The final predictions are not based on any single tree but rather on the entire forest. The use of the entire forest rather than an individual tree helps avoid overfitting the model to the training dataset, as does the use of both a random subset of the training data and a random subset of explanatory variables in each tree that constitutes the forest [24]. The available publications suggest the predominant transmission of EHV by direct contact and by environment (including air, water, and fomites) [25]. Given this information, and considering a lack of publications studying spatio-temporal patterns of EHV-1 spread, we chose to test a most generic set of variables at district level, including:

1. Landscape and climatic factors that may affect virus survival in the environment and provide short-distance airborne spread: mean yearly air temperature, mean yearly precipitation, altitude, average wind speed in January and July (as representatives of the coldest and warmest seasons in Kazakhstan, respectively), and maximum green vegetation fraction (MGVF) as a representative of the intensity of the green cover.
2. Sociodemographic proxies that may influence the intensity of between-farm contacts include horse population density, general population density, density of populated places, number of horse farms, and the number of objects targeted for surveillance (OTS). OTS refer to infrastructure entities that can act as focal points and potential conduits for the transmission of infectious animal diseases and therefore require continuous epidemiological monitoring. These entities include slaughterhouses, meat processing facilities, and animal markets. A database of such objects was compiled in Kazakhstan between 2019 and 2022. In our previous study, we demonstrated a statistically significant association between the presence of these objects and the intensity of the animal disease situation [26].
3. Transportation factors that additionally influence the between-farm contacts and stipulate a potential virus transportation from infected areas (including bordering countries): road length, road density, bordering with another country, and presence of an arterial road passing through the district.

We removed four modeling districts with zero horse population and with high population density (city areas), so the total number of modeled districts was 172. The forest-based classification and regression model was trained on the EHV-1 data using 50,000 trees with four randomly sampled variables per decision tree. Twenty-five percent of input data was set aside for validation, which was performed in 500 runs. A best trained model was used for prediction that forecasted the infectious status of each RK district based on the predictors' values.

### 2.5. Variables' Data Source and Processing

Climatic factors such as air temperature and precipitation were extracted from ERA5 reanalysis datasets for 2017–2022 available from Copernicus Climate Data Store (https://cds.climate.copernicus.eu/datasets), while altitude were sourced from the WorldClim web portal [27]. MGVF data were retrieved from the NASA Modis data portal (https://modis.gsfc.nasa.gov/data/) [28]. Population density was obtained from the Gridded Population of the World (GPW), v4 dataset available at the SEDAC data portal [29]. Road length and density were calculated in GIS using the data obtained from the global road network provided by Esri (https://www.arcgis.com/home/item.html?id=ed3188a199a84ce2bf7edfb97b241ec8).

**Figure 2:**
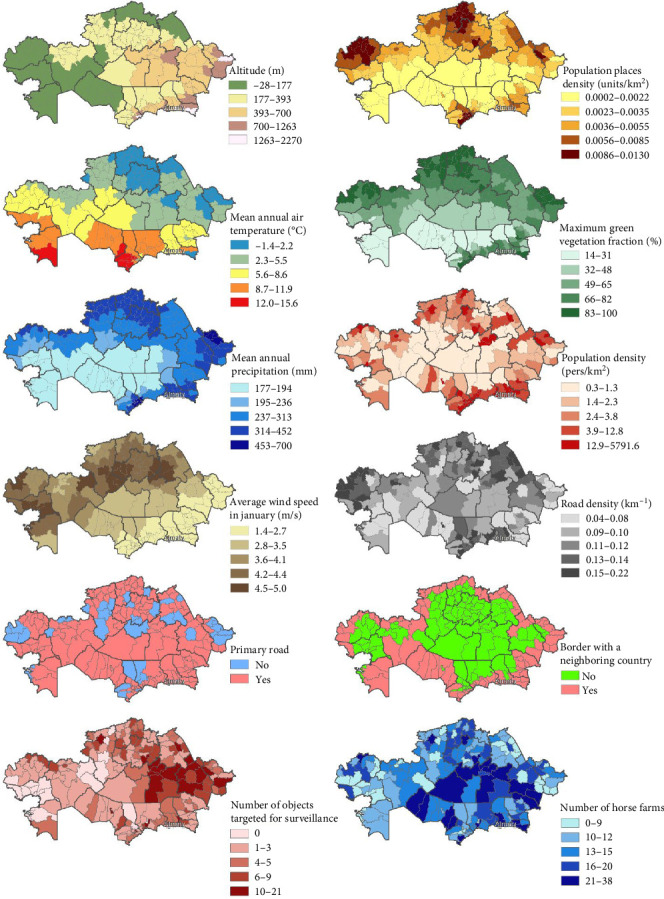
Spatial distribution of explanatory variables used for EHV-1 modeling in Kazakhstan.

Despite the forest-based model is known to be less sensitive to potential multicollinearity of explanatory variables, we preferred to remove those factors that demonstrated a significant correlation (|*r*|≥0.7) with other variables. All variables were tested using a Spearman rank correlation approach. From each pair of correlated variables, we removed that one, which showed a higher correlation with other factors.  Table 2 presents the final set of variables used for modeling, their descriptions, and ranges. The variables are visualized in Figure 2.

### 2.6. Software

Visualization, processing of geospatial variables, and forest-based modeling were conducted by using the geographic information systems ArcGIS Desktop 10.8.2 and ArcGIS Pro 3.1.0 (Redlands, CA, USA). The multicollinearity of variables was tested in the statistically-oriented software environment R using the *corrplot* package [30].

## 3. Results

**Figure 3:**
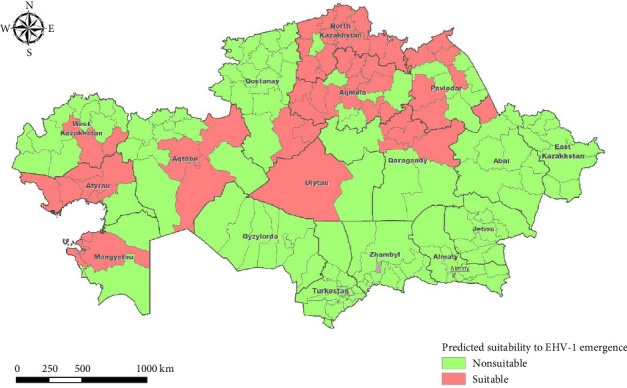
Predicted suitability of Kazakhstan districts to EHV-1 emergence.

Modeling by the forest-based classification and regression model demonstrated the predominated significance of the average wind speed in January, road density, number of horse farms, number of livestock-related facilities targeted for surveillance, followed by the rest of tested variables. The presence of an arterial road and sharing border with a neighboring country were the least significant variables (Table 3). The mean training accuracy was 0.79, while the validation accuracy was 0.73. The map of predicted suitability to EHV-1 infection is provided in Figure 3.

## 4. Discussion

The conducted modeling of EHV-1 infection suitability in the RK was aimed at revealing the primary and most generic environmental and socio-demograpfic conditions associated with the affected municipalities of the Republic. The choice of the forest-based modeling method is justified by a small number of infected districts (*n* = 10), as well as the absence of detailed epidemiological information about the number of diseased animals, which could allow calculating the prevalence and applying other regression analysis methods.

There are virtually no publications on the spatial analysis specific to the equine rhinopneumonia EHV-1 virus. The limited availability of information on documented EHV-1 cases in Kazakhstan restricts the possibility of conducting a detailed spatio-temporal analysis, leaving only the use of infected districts as the response variable. In this study, we have attempted to apply the well-documented method of forest-based modeling. This approach is known to be less sensitive to variables' multicollinearity and scale, and to capture both linear and nonlinear relationships. Its drawbacks include mainly model complexity, increased calculation time, and lack of clear interpretation of returned variables' importance metrics: unlike a traditional regression approach, the forest-based model provides a relative importance only and do not offer full visibility into the coefficients.

In the set of potential explanatory variables, we included a generic range of factors covering both landscape-climatic and socio-demographic conditions. Some recent research suggest that EHV-1 virus may be transmitted via environment that justified inclusion of environmental factors in our analysis [25, 31]. Thus, average wind speed may be associated with the probability of airborne virus transmission, while air temperature, precipitation, green cover, and altitude may influence the virus's survival in the environment. We have introduced the factors proxying the concentration of population and susceptible animals as well as the intensity of transport and economic links that may represent an area's susceptibility to the virus transmission by direct contact of via fomites.

Our hypothesis was that the EHV-1-infected districts would be found to be crossed by arterial roads leading from neighboring countries or would have a common border with other countries. This would suggest a potential introduction of virus by transportation from other countries. Surprisingly, these two factors demonstrated the least importance in the set of other variables. A conclusion can be made here (though requiring further investigation) that a long-distance transportation does not play a major role in the spread of EHV-1 virus. In part, it can be confirmed by a fact of relatively short EHVs survival in fomites with which it can be potentially transmitted [32, 33].

In contrast, the factors proxying the concentration of horse population and the intensity of transportation (horse population density, number of horse farms, and road density), have demonstrated a biggest influence on the infection status of districts suggesting a predominately short-distance transmission of the virus, which corresponds well to the current knowledge of EHV-1 epidemiology [25]. The areas that were identified by the model as vulnerable, mainly located in the north and south-west parts of the country with high population density, high precipitation, but lower average temperatures. Highest in Kazakhstan concentration of population places can be found in the northern areas. These regions are characterized by the presence of a large number of small-scale rural horse breeding farms with a low level of biosafety, as well as a developed transportation network and high economic activity, which can contribute to the transmission of the virus. A peculiarity of horse-breeding seasonality, when horses are kept within farms (or at closed pastures) during unfavorable weather conditions promoting more intensive horse-to-horse contacts, can be seen as another possible explanation of the obtained model results [34, 35]. A higher wind speed in January was found to be strongly associated with the probability of EHV-1 emergence, which either may be related to the peculiarities of climate in northern regions of Kazakhstan, where strong winds are typical all over the year, or may suggest an effect of EHV-1 virus airborne transmission [31]. Another factor found to significantly contribute to the observed distribution of EHV-1 infection was the number of OTS. These are facilities that serve as points of contact between animal populations and between animals and humans, thereby increasing the likelihood of disease transmission and necessitating enhanced surveillance. Examples of such OTS include slaughterhouses, meat processing plants, livestock markets, and veterinary laboratories. In our previous study, the presence of OTS in a district was found to be associated with the brucellosis infection status of that district [33]. According to our hypothesis, the presence of OTS implies an increased volume of animal movements both within the district and from adjacent areas, as well as a higher risk of contamination through personnel and vehicles. Based on the results of our analysis, it can be concluded that further modeling efforts should be put into collecting country-wise data containing an information on the number of infected and susceptible animals as well as location of infected farms, which would allow applying prevalence-based modeling approaches. The obtained results may be of importance to the national veterinary service by indicating potentially vulnerable districts, where EHV-1 monitoring efforts should be strengthened.

## Figures and Tables

**Table 1:** Yearly distribution of the number of EHV-1-infected farms in Kazakhstan, 2017–2022.

Year	2017	2018	2019	2020	2021	2022	2023	2024
Number of EHV-1-infected farms	1	4	2	4	6	12	2	3

**Table 2:** List of variables used in forest-based modeling for EHV-1 in Kazakhstan.

Variable	Description	Median values and range
Alt	Altitude above sea level (m)	250 (−136–5983)
Mean_temp	Mean annual air temperature (°C)	4.4 (−1.4–15.6)
Mean_prec	Mean annual precipitation (mm)	297 (117–700)
Wind_1	Average wind speed in January (m/s)	3.8 (1.4–5.0)
Border	Presence of a border with neighboring country	Yes/no
Main_road	Presence of an arterial road passing through the district	Yes/no
Pop_place_dens	The density of populated places (units/km)^2^	0.004 (0.0001–0.01)
Horse_dens	The density of horse population, (head/km)^2^	1.4 (0.2–34.1)
MGVF	Maximum green vegetation fraction (%)	75 (14–100)
Pop_dens	Population density (persons/km)^2^	2.9 (0.3–307.0)
Road_dens	Road density (km)^−1^	0.09 (0.05–0.18)
Horse_farms_num	Number of horse farms per district	13 (1–38)
Epid_obj	Number of objects targeted for surveillance per district	3 (0–21)

**Table 3:** Relative contribution of variables into the forest-based classification and regression model of EHV-1 virus in Kazakhstan.

Variable	Percent contribution
Average wind speed in January	16
Road density	9
Number of horse farms	8
Number of objects targeted for surveillance	8
Altitude	8
Mean air temperature	7
Mean precipitation	7
Population density	7
MGVF	7
Populated places density	7
Horse population density	7
Presence of main road	6
Presence of country border	3

*Note:* The percent contribution represents a percentage of the total sum of Gini coefficients (the Gini coefficient is a metric that quantifies the reduction in uncertainty at a decision tree node when a particular feature is used to split the data).

## Data Availability

The data on EHV-1 infection status of the districts of the Republic of Kazakhstan can be obtained from the corresponding author upon a reasonable request.

## References

[1] Berdimurat N. (2015). Cost Accounting in the Horse Breeding Industry of Kazakhstan Improving due to International Standards. *Economic Annals-XXI*.

[2] Kulasheva A. Horse Breeding in Kazakhstan: Development Prospects and Current Realities. https://world-nan.kz/en/blogs/konevodstvo-v-kazakhstane-perspektivy-razvitiya-i-segodnyashnie-realii.

[3] Uskenov B., Kairat Z., Iskhan T. S. (2023). Creation of Smart Farms in the Herd Horse Breeding of Kazakhstan (Results of Using Trackers). *OnLine Journal of Biological Sciences*.

[4] Reed S. M., Toribio R. E. (2004). Equine Herpesvirus 1 and 4. *Veterinary Clinics of North America: Equine Practice*.

[5] Sutton G., Garvey M., Cullinane A. (2019). Molecular Surveillance of EHV-1 Strains Circulating in France During and After the Major 2009 Outbreak in Normandy Involving Respiratory Infection, Neurological Disorder, and Abortion. *Viruses*.

[6] Sutton G., Normand C., Carnet F. (2021). Equine Herpesvirus 1 Variant and New Marker for Epidemiologic Surveillance, Europe, 2021. *Emerging Infectious Diseases*.

[7] Phillips S. J., Anderson R. P., Schapire R. E. (2006). Maximum Entropy Modeling of Species Geographic Distributions. *Ecological Modelling*.

[8] Kydd J. H., Townsend H. G. G., Hannant D. (2006). The Equine Immune Response to Equine Herpesvirus-1: The Virus and its Vaccines. *Veterinary Immunology and Immunopathology*.

[9] Gonzalez-Medina S., Newton J. R. (2015). Equine Herpesvirus-1: Dealing Practically but Effectively With an Ever Present Threat. *Equine Veterinary Journal*.

[10] Yurov К. P., Zablotskyi V. Т., Kosminkov N. E. (2010). *Viral Diseases of Horses*.

[11] Tsybanov S. J., Tsybanova L. Y., Kadykoyev M. I., Kalabekov R. T. (2000). Identification of Equine Rhinopneumonia Virus by PCR.

[12] Oladunni F. S., Horohov D. W., Chambers T. M. (2019). EHV-1: A Constant Threat to the Horse Industry. *Frontiers in Microbiology*.

[13] Patel J. R., Heldens J. (2005). Equine Herpesviruses 1 (EHV-1) and 4 (EHV-4)—Epidemiology, Disease and Immunoprophylaxis: A Brief Review. *The Veterinary Journal*.

[14] Agerholm J. S., Klas E. M., Damborg P., Borel N., Pedersen H. G., Christoffersen M. (2021). A Diagnostic Survey of Aborted Equine Fetuses and Stillborn Premature Foals in Denmark. *Frontiers in Veterinary Science*.

[15] Neustroyev М. P., Tarabukina N. P., Reshetnikov А. D., Kokolova L. M. (2018). Regional Epizootology of Infectious and Invasive Diseases of Horses. *Siberian Bulletin of Agricultural Science*.

[16] Tong P., Duan R., Palidan N. (2022). Outbreak of Neuropathogenic Equid Herpesvirus 1 Causing Abortions in Yili Horses of Zhaosu, North Xinjiang, China. *BMC Veterinary Research*.

[17] Allen G. P., Bolin D. C., Bryant U. (2008). Prevalence of Latent, Neuropathogenic Equine Herpesvirus-1 in the Thoroughbred Broodmare Population of Central Kentucky. *Equine Veterinary Journal*.

[18] Pusterla N., Barnum S., Miller J. (2021). Investigation of an EHV-1 Outbreak in the United States Caused by a New H752 Genotype. *Pathogens*.

[19] Shalgynbayev E. К., Kospanova М. N., Ryabinnikova А. I., Omarova Z. D., Orynbayev M. B. (2014). Monitoring, Isolation, Identification and Cultivation of Equine Herpesvirus across the Territory of the Republic of Kazakhstan.

[20] Breiman L. (2001). Random Forests. *Machine Learning*.

[21] Breiman L., Friedman J. H., Olshen R. A., Stone C. J. (2017). *Classification and Regression Trees*.

[22] Ao Y., Li H., Zhu L., Ali S., Yang Z. (2019). The Linear Random Forest Algorithm and its Advantages in Machine Learning Assisted Logging Regression Modeling. *Journal of Petroleum Science and Engineering*.

[23] Dureh N., Ueranantasan A., Eso M. A Comparison of Multiple Linear Regression and Random Forest for Community Concern of Youth and Young Adults Survey. *Songklanakarin Journal of Science and Technology*.

[24] Esri. ArcGIS Pro Help (2024). How Forest-Based Classification and Regression Works. https://pro.arcgis.com/en/proa-app/3.1/tool-reference/spatial-statistics/how-forest-works.htm.

[25] Dayaram A., Seeber P. A., Greenwood A. D. (2021). Environmental Detection and Potential Transmission of Equine Herpesviruses. *Pathogens (Basel, Switzerland)*.

[26] Mukhanbetkaliyeva A. A., Kadyrov A. S., Mukhanbetkaliyev Y. Y. (2024). Identification and Mapping of Objects Targeted for Surveillance and Their Role as Risk Factors for Brucellosis in Livestock Farms in Kazakhstan. *Geospatial Health*.

[27] Fick S. E., Hijmans R. J. (2017). WorldClim 2: New 1km Spatial Resolution Climate Surfaces for Global Land Areas. *International Journal of Climatology*.

[28] Broxton P. D., Zeng X., Scheftic W., Troch P. A. (2014). A MODIS-Based Global 1-Km Maximum Green Vegetation Fraction Dataset. *Journal of Applied Meteorology and Climatology*.

[29] Center For International Earth Science Information Network-CIESIN-Columbia University (2018). *Gridded Population of the World, Version 4 (GPWv4): Population Density, Revision 11*.

[30] Wei T., Simko V. (2024). R Package ’Corrplot’: Visualization of a Correlation Matrix. https://github.com/taiyun/corrplot.

[31] Derbal S. (2021). Epidemiology of EHV-1 and EHV-4 Infections: A Review. *International Journal of Veterinary Science and Research*.

[32] Vandenberghe E., Boshuizen B., Delesalle C. J. G. (2021). New Insights Into the Management of an EHV-1 (Equine Hospital) Outbreak. *Viruses*.

[33] Lunn D. P., Davis-Poynter N., Flaminio M. J. (2009). Equine Herpesvirus-1 Consensus Statement. *Journal of Veterinary Internal Medicine*.

[34] Naimanov D. K., Turabayev A. T., Bakhtybayev G. T., Seleuova L. A. (2018). Herd Horse Breeding (Tabunnoe Konevodstvo) (in Russian). https://ksu.edu.kz/files/TB/book/vet/seleuova_l_a_na_zamenu_praktikum_tabunnoe_konevodstvo.pdf.

[35] Alimaev I. (2019). How we Need to Develop a Transhumant Animal Husbandry. https://agrosektor.kz/livestock/kak-nam-razvivat-otgonnoe-zhivotnovodstvo.html?ysclid=llj63vz49o587533654.

